# Upper Extremity Neurovascular Injuries in Collegiate Athletes: Sex, Race, and Return to Sport

**DOI:** 10.1177/23259671251405805

**Published:** 2026-02-20

**Authors:** David M. Heath, Stephanie D. Talutis, Autreen Golzar, Jesus G. Ulloa, Hugh A. Gelbert, Sharon L. Hame

**Affiliations:** * Department of Orthopaedics, University of Texas Health Science Center at San Antonio, San Antonio, Texas, USA; † Department of Vascular Surgery, Boston Medical Center, Boston, Massachusetts, USA; ‡ David Geffen School of Medicine at UCLA, Los Angeles, California, USA; ‖ Department of Vascular Surgery at UCLA, Los Angeles, California, USA; ¶ Department of Orthopaedic Surgery at UCLA, Los Angeles, California, USA; Investigation performed at the University of California, Los Angeles, Los Angeles, California, USA

**Keywords:** general sports trauma, epidemiology, cervical spine, peripheral nerve injuries, upper extremity

## Abstract

**Background:**

Upper extremity neurovascular injuries (UENVIs) are a significant yet understudied aspect of sports medicine. Although distinct UENVIs have been independently studied, no report describes the incidence of UENVIs across all collegiate sports.

**Purpose:**

To analyze the occurrence, demographics, treatment modalities, and outcomes associated with UENVIs across various sports in collegiate athletes using data from the Pac-12 Health Analytics Program database.

**Study Design:**

Case series; Level of evidence, 4.

**Methods:**

The Pac-12 Health Analytics Program database was queried for brachial plexus injuries and sports-related UENVIs over a 7-year period (2016-2022). UENVIs were subdivided into neurologic neck injury, transient brachial plexus injury, and thoracic outlet syndrome. Statistical analysis consisted of the chi-square test for categorical variables and analysis of variance for continuous variables. Significance was defined as *P* < .05.

**Results:**

In total, 15,609 athletes with injuries across 21 sports were identified, of whom 213 experienced UENVIs. Of the athletes, 155 (72.8%) were male and 58 (27.2%) were female. Football players comprised the largest number of UENVI cases. When football players were excluded from the analysis, female athletes had a higher incidence of UENVIs at 61.7% compared to males at 38.3%. Most players were White (50.8%) or Black (23%). Among all athletes in each sport, the highest rates of UENVIs were observed in wrestling (3.9%), football (3.6%), and tennis (2.6%). Of the athletes, 95.5% with UENVIs returned to their previous level of activity or reported no interference with their sport from the injury.

**Conclusion:**

Most UENVIs in collegiate athletes are transient and self-limited. This is the first study to report on sex and race distribution for UENVIs. Many athletes return to sport at a similar or higher level.

Upper extremity neurovascular injuries (UENVIs) include conditions involving damage or trauma to the nerves and blood vessels in the upper limb, predominantly affecting the brachial plexus or subclavian artery. UENVIs represent an understudied domain in sports medicine, especially among collegiate athletes. The most common UENVIs can be broadly categorized as neurologic neck injuries, transient brachial plexus injuries, and thoracic outlet syndrome (TOS).

Neurologic neck injuries are peripheral nerve injuries that involve compression of cervical nerve roots (eg, C5-C6, C8-T1) and present with symptoms such as neck pain, radicular pain, and sensory deficits in the corresponding dermatomes.^1,8^ These injuries have transient symptoms, including neck pain and nerve root dysesthesia, and are typically self-limiting, with a low likelihood of permanent career-ending consequences. In the existing literature, these pathologies are infrequently reported in athletes, and the epidemiology and sport-specific burden of neurologic neck injuries remain poorly defined.^11,27^

Transient brachial plexus injuries, known as burners or stingers, typically involve the upper trunk of the brachial plexus (C5-C6) and are characterized by sudden, sharp pain radiating from the neck into the arm, often accompanied by a burning sensation and temporary weakness or numbness.^28^ Stingers are common in contact sports like football and rugby and usually result from downward traction of the shoulder due to a forceful impact.^2^ Athletes with a single isolated stinger that resolves rapidly may be considered for return to play in the same game, while recurrent stingers or persisting symptoms may require withholding the athlete from play and further evaluation.^2^ They have been identified as the most common cause of upper extremity neurologic injury in elite American football athletes, accounting for 46% of injuries in the National Football League and 50% to 65% in collegiate football.^26,28^ Studies in National Collegiate Athletic Association (NCAA) football identified player contact as the mechanism of injury for 93.0% of stingers, mostly from tackling (36.7%) and blocking (25.8%).^14^ Chung et al^6^ found the incidence of cervical spine injuries in collegiate football players to be 2.91 per 10,000 athletic events, which included practice and competition. Of these, stingers accounted for 1.87 per 10,000 athletic events, followed by cervical strains at 0.80 per 10,000 athletic events. Despite the high rate of injury within professional football, stingers are likely underreported because of their transient nature, strong desire by athletes to return to play, and the perception of possible career implications if athletes remove themselves from participation.^18^ Additionally, burners are a common contact sport injury resulting from trauma to the neck and shoulder, occurring most often in football and rugby but also reported in wrestling, gymnastics, hockey, basketball, boxing, and weightlifting.

TOS involves compression of the brachial plexus (C8-T1) or the subclavian vessels in the thoracic outlet.^7,19,26^ Neurogenic TOS is by far the most common type resulting from brachial plexus compression, accounting for >95% of cases; less common subtypes include arterial TOS and venous TOS, predominantly involving compression of the subclavian artery and vein, respectively.^3^ Athletes involved in repetitive overhead movements face an elevated risk of developing TOS due to actions increasing compression of neurovascular structures, such as arm abduction, pulling shoulders inferiorly and posteriorly, muscle swelling, trauma, exercise, or hypertrophy.^23^ However, there is no consensus for diagnostic and therapeutic criteria, and treatment algorithms may vary widely among clinicians.^12,13^ Options in management leading to successful return to play may include nonsteroidal anti-inflammatory drugs, intensive physical therapy, botulinum toxin injections, or first rib resection and scalenectomy.^5,9,10,17^ The success of nonoperative and surgical options in studies including NCAA and Major League Baseball athletes ranges from 70% to 90%, with most athletes returning to competition within 6 months to 1 year.^4,24,29-32^

Although distinct UENVIs have been independently studied in the context of college and professional football and baseball, no studies have reported the incidence of UENVIs across NCAA collegiate sports. Through the utilization of the Pac-12 Health Analytics Program database, we aim to identify the occurrence, demographics, sports distribution, treatment modalities, and outcomes associated with UENVIs in collegiate athletes.

## Methods

### Data Collection

The Pac-12 Health Analytics Program database was queried for the occurrence of brachial plexus injuries and UENVIs over a 7-year period from 2016 to 2022. Demographics, sport, treatment, and outcome were recorded. The study population consisted of Pac-12 collegiate athletes during this observation period who consented to the use of their deidentified demographic and illness information for research. This study was considered exempt from approval by the local institutional review board.

The Pac-12 Sports Injury Research Archive contains the following data for each injury or illness event: deidentified athlete identification number, sex, age, race, sport, body part affected, event year, type of injury or illness determined by an orthopaedic surgeon, mechanism of injury, in-season versus off-season timing, event setting, days from injury to examination, requirement for physician encounter, requirement for diagnostic testing, requirement for surgery or other treatment, and return-to-play data. The initial injury assessment is typically performed by a certified athletic trainer. This is followed by evaluation by a sports medicine physician or orthopaedic surgeon, who determines the final diagnosis. The database is updated by physicians and athletic trainers over the course of each illness or injury. The completeness of the database has been evaluated, with data quality control measures in place to ensure accuracy and value. A study by Robell et al^25^ highlighted that the Pac-12 Health Analytics Program, which includes this database, has implemented rigorous data quality control processes to improve data accuracy and completeness. Additionally, the completeness of injury data provided by medical teams and local organizing committee physicians averaged 95.8% in similar surveillance systems.

Deidentified player data from the Pac-12 Sports Injury Research Archive were abstracted for analysis. The database was queried for UENVIs. UENVIs were categorized into (1) neurologic neck injury, (2) transient brachial plexus injury, and (3) thoracic outlet syndrome.

Athletes were grouped into the following sports: baseball, basketball, football, gymnastics, lacrosse, rowing, soccer, softball, swimming, tennis, track and field, volleyball, water polo, and wrestling. Any athlete not fitting into these categories was placed under the unique sports category.

### Statistical Analysis

Statistical analysis was performed using Microsoft Excel Version 16.49. Categorical variables were analyzed using chi-square testing. Analysis of variance was used to analyze continuous variables. Significance was defined as *P* < .05.

## Results

A total of 15,609 athletes with injuries across 21 sports were identified. No athletes were excluded from the database query. The average age among all collegiate athletes was 23.0 ± 3.1 years. The breakdown of athletes by sport and the percentage who developed UENVIs is shown in Table 1. In total, 213 athletes with UENVIs were identified. The mean age at the time of injury was 19.9 ± 2.4 years. The UENVI cohort comprised 155 (72.8%) male athletes and 58 (27.2%) female athletes. Most athletes were White (50.8%) or Black (23%). Hawaiians/Pacific Islanders comprised 9% of the cohort, 3% were Hispanic/Latino, and 2.5% were Indian/Native Alaskan. Additional demographics are outlined in Table 2.

**Table 1:** Injury by Sport*^*a*^*

Sport	Number of Athletes in Sport (n = 15,609)	Number of Athletes With UENVIs (n = 213)	Percentage of Athletes With UENVIs
Baseball	1089	7	0.6
Basketball	919	9	1.0
Cross country	443	0	0.0
Diving	96	0	0.0
Field hockey	83	0	0.0
Football	3341	119	3.6
Golf	461	0	0.0
Gymnastics	364	2	0.5
Lacrosse	414	1	0.2
Rowing	1435	17	1.2
Skiing	198	0	0.0
Soccer	1108	3	0.3
Softball	454	7	1.5
Swimming	893	7	0.8
Tennis	463	12	2.6
Track and field	1631	5	0.3
Unique sports	680	4	0.6
Volleyball	867	8	0.9
Water polo	438	3	0.7
Wrestling	232	9	3.9

a UENVI, upper extremity neurovascular injury.

**Table 2:** Demographics*^*a*^*

Characteristic	Athletes in Sport (n = 15,609)	All Sports-Related UENVIs (n = 213)	Thoracic Outlet Syndrome (n = 73)	Transient Brachial Plexus Injuries (n = 126)	Neurologic Neck Injury (n = 14)	*P* Value
Sex						
Female	44.1	27.2	61.6	8.7	14.3	**<.001**
Male	55.9	72.8	38.4	91.3	85.7	
Age at Injury, mean ± SD, y	23.0 ± 3.1	19.9 ± 2.4	19.3 ± 3.5	20.2 ± 1.6	20.3 ± 0.9	**.024**
Race						**<.001**
Black	7.8	23.0	7.0	34.3	11.1	
Hispanic/Latino	2.0	3.3	4.7	0.0	22.2	
White	26.4	50.8	74.4	38.6	33.3	
Native						
Hawaiian/Pacific Islander	1.4	9.0	0.0	12.9	22.2	
American Indian/Alaska						
Native	0.17	2.5	0.0	2.9	11.1	
Two or more races	3.3	8.2	9.3	8.6	0.0	
Unknown	56.8	3.3	4.7	2.9	0.0	
Sports						
Baseball	7.0	3.3	8.2	0.8	0.0	
Basketball	5.9	4.2	2.7	5.6	0.0	
Football	21.4	55.9	8.2	81.0	78.6	
Gymnastics	2.3	0.9	2.7	0.0	0.0	
Lacrosse	2.7	0.5	0.0	0.8	0.0	
Rowing	9.1	8.0	23.3	0.0	0.0	
Soccer	7.1	1.4	0.0	1.6	7.1	
Softball	2.9	3.3	8.2	0.8	0.0	
Swimming	5.7	3.3	9.6	0.0	0.0	
Tennis	3.0	5.6	16.4	0.0	0.0	
Track and field	10.4	2.3	5.5	0.8	0.0	
Unique sports	4.4	1.9	1.4	1.6	7.1	
Volleyball	5.6	3.8	8.2	1.6	0.0	
Water polo	2.8	1.4	4.1	0.0	0.0	
Wrestling	1.5	4.2	1.4	5.6	7.1	

a Values are presented as percentages unless otherwise indicated. UENVI, upper extremity neurovascular injury. Bold values indicate statistical significance (*P* < .05).

When categorizing UENVIs, we identified 14 (6.5%) neurologic neck injuries, 126 (59.2%) transient brachial plexus traction injuries, and 73 (34.3%) cases of TOS. Among all athletes in each sport, the highest rates of UENVIs were observed in wrestling (3.9%), football (3.6%), and tennis (2.6%) (Table 1). Football accounted for 55.9% (119/213) of all UENVIs while representing 21.4% (3341/15,609) of athletes in the cohort.

Most injuries were sports related (90.1%), including 78.1% of TOS, 96.0% of transient brachial plexus injuries, and 100% of neurologic neck injuries (*P* < .001). In all, 78.6% of UENVIs were new injuries, 81.3% were acute injuries, and 18.7% were chronic/overuse injuries. UENVIs resulting from contact mechanisms were reported in 91.9% of transient brachial plexus injuries but in only 4.3% of TOS cases. Overall, 47.3% of athletes did not miss any time from their sport. The mechanism of injury was contact in 61.5% of athletes. Injuries occurred more often in practice than in any other injury event. Injury type, onset of symptoms, missed time, mechanism, and injury event for each UENVI type are described in Table 3.

**Table 3:** Injury Type*^*a*^*

Characteristic	All Sports-Related UENVIs (n = 213)	Thoracic Outlet Syndrome (n = 73)	Transient Brachial Plexus Injuries (n = 126)	Neurologic Neck Injury (n = 14)	*P* Value
No. of injuries, mean ± SD	1.1 ± 0.4	1.0 ± 0.2	1.2 ± 0.5	1.0 ± 0.0	**.013**
Injury type					.34
New injury	78.6	73.2	81.6	78.6	
Preexisting	2.4	5.6	0.8	0.0	
First-time recurrence	9.5	12.7	8.0	7.1	
Multiple recurrences	9.5	8.5	9.6	14.3	
Onset of symptoms					**<.001**
Acute	81.3	52.1	96.8	92.9	
Chronic/overuse	18.7	47.9	3.2	7.1	
Prognosis					**<.001**
No missed time	47.3	42.6	51.2	35.7	
<1 week	16.6	4.4	20.3	42.9	
1-4 weeks	15.1	16.2	15.4	7.1	
4-8 weeks	1.0	2.9	0.0	0.0	
8-24 weeks	2.9	7.4	0.8	0.0	
>24 weeks	1.0	2.9	0.0	0.0	
Unknown	16.1	23.5	12.2	14.3	
Mechanism					**<.001**
No contact	3.8	10.0	0.8	0.0	
Contact	61.5	4.3	91.9	78.6	
Throwing	6.3	15.7	1.6	0.0	
Repetitive motion	7.2	20.0	0.8	0.0	
Nonspecific	5.3	12.9	0.8	7.1	
N/A	13.5	35.7	2.4	0.0	
Unknown mechanism	1.4	1.4	0.0	14.3	
Injury event					**<.001**
Competition	26.9	5.7	37.9	35.7	
Practice	51.0	40.0	56.5	57.1	
Weight room	2.4	2.9	1.6	7.1	
Strength training	1.0	2.9	0.0	0.0	
Voluntary workout	1.0	2.9	0.0	0.0	
Nonsports related	1.4	2.9	0.8	0.0	
N/A	16.3	42.9	3.2	0.0	

a Values are presented as percentages unless otherwise indicated. N/A, not applicable. Bold values indicate statistical significance (*P* < .05). UENVI, upper extremity neurovascular injury.

As a result of the injury, 55.4% of UENVIs required physician evaluation, with 35.2% requiring diagnostic testing. Physician evaluation was more common among TOS (69.9%) and neurologic neck injuries (78.6%) compared to transient brachial plexus injuries (44.4%) (*P* < .001). Further diagnostic test distribution followed a similar pattern, with 46.6% of TOS and 64.3% of neurologic neck injuries undergoing diagnostic tests compared with 25.4% of transient brachial plexus injuries (*P* < .001). Athletes with TOS required more procedures (6.8%) and surgery (13.7%) (*P* < .001) and underwent more treatments compared to other groups (*P* < .001) (Table 4).

**Table 4:** Treatment*^*a*^*

Characteristic	All UENVIs (n = 213)	Thoracic Outlet Syndrome (n = 73)	Transient Brachial Plexus Injuries (n = 126)	Neurologic Neck Injury (n = 14)	*P* Value
Days from injury to exam	11.1 ± 76.0	19.5 ± 87.1	7.5 ± 73.3	0.4 ± 0.9	.49
Injury management					
Physician evaluation	55.4	69.9	44.4	78.6	**<.001**
Medication prescribed	8.0	6.8	8.7	7.1	.89
Diagnostic test	35.2	46.6	25.4	64.3	**<.001**
Procedure	2.8	6.8	0.8	0.0	**.036**
Surgery	4.7	13.7	0.0	0.0	**<.001**
Mean number of treatments	10.6 ± 18.9	20.4 ± 27.2	5.5 ± 9.4	4.4 ± 6.7	**<.001**

a Values are presented as percentages unless otherwise indicated. Bold values indicate statistical significance (*P* < .05). UENVI, upper extremity neurovascular injury.

Injuries resolved in 80.8% of TOS, 97.6% of transient brachial plexus injuries, and 100% of neurologic neck injuries (*P* < .001). Overall, 95.5% of athletes returned to their previous level of activity or reported no interference with their sport from the injury. Of the athletes, 1.0% returned with restrictions, 2.0% did not return to play, and 1.5% were lost to follow-up (ie, left campus) before return-to-play information was gathered. Of athletes initially missing time from their sport, 37.9% of TOS, 43.0% of transient brachial plexus injuries, and 28.6% of neurologic neck injuries returned fully to their previous activity levels. Of those with TOS, 3.0% returned to sport with restrictions, but all players in this cohort returned to play. While no players returned with restrictions in the other cohorts, 2.5% with transient brachial plexus injuries and 7.1% with neurologic neck injuries did not return to play. Mean days to clearance across all UENVIs were 39.0 ± 170.7 and highly varied among subcategories. Time to clearance was longest among those with TOS at 92.5 ± 231.9 days (*P* = .003) (Table 5).

**Table 5:** Outcomes*^*a*^*

Characteristic	All UENVIs (n = 213)	Thoracic Outlet Syndrome (n = 73)	Transient Brachial Plexus Injuries (n = 126)	Neurologic Neck Injury (n = 14)	*P* Value
Injury resolved	92.0	80.8	97.6	100.0	**<.001**
Performance					.068
Injury did not interfere with sport	55.2	54.5	54.5	64.3	
Returned to previous activity level	40.3	37.9	43.0	28.6	
Returned to restricted activity level	1.0	3.0	0.0	0.0	
Unable to return to previous activity level	2.0	0.0	2.5	7.1	
Left campus prior to returning to play	1.5	4.5	0.0	0.0	
Mean days to clearance	39.0 ± 170.7	92.5 ± 231.9	7.1 ± 118.9	46.3 ± 125.3	**.003**

a Values are presented as percentages unless otherwise indicated. Bold values indicate statistical significance (*P* < .05). UENVI, upper extremity neurovascular injury.

## Discussion

UENVIs may result in morbidity among collegiate athletics, yet comprehensive studies on the incidence, demographics, treatment, and outcomes of these injuries across various sports are lacking. The purpose of our study was to address this gap in literature by analyzing data from the Pac-12 Health Analytics Program database. We identified 213 cases of UENVIs among 15,609 collegiate athletes, with football emerging as the sport with the highest incidence of UENVI injuries. To our knowledge, this is the first study in collegiate athletes to report on sex and race distribution in UENVI. Male athletes accounted for 72.8% of all neurovascular injuries despite making up only 53% of the Division I NCAA athletic population.^22^ This can largely be attributed to participation in football, which alone accounted for 55.9% (119/213) of all UENVIs while only accounting for 21.4% (3341/15,609) of athletes. Notably, if football players are excluded from analysis, female athletes have a higher incidence of UENVIs at 61.7% (58/94) of athletes compared to male athletes at 38.3% (36/94). When analyzing the types of injuries, female athletes had a higher incidence of TOS (61.6%), which may be largely attributed to a higher incidence of female athletes with neurogenic TOS.^16,20^ TOS is characterized by compression of neurovascular structures at the thoracic outlet and may present as arm pain, swelling, fatigue, paresthesias, weakness, or discoloration depending on the affected structure.^24^ Severe forms can include limb-threatening ischemia or irreversible neurovascular damage with loss of function. TOS may be attributed to both congenital and acquired causes, such as a cervical rib or acute injury in the setting of chronic compression.^21^ TOS is relatively understudied in athletes but has been shown to predominantly affect female athletes as well as in the general population.^21^ A study of 41 competitive athletes, 66 with neurogenic TOS and 34% with Paget-Schroetter syndrome, found an 85% return-to-play rate and a recurrence rate of approximately 10%.^4^

When considering an athlete's race, 50.8% (108/213) of players with UENVI in this study were White and 23% (49/213) were Black. White players account for 55% of Division I NCAA collegiate athletes, while Black players account for 20% and are more likely to participate in contact sports.^22^ Most cases of UENVIs in our study were acute, transient brachial plexus injuries from direct player contact. Most of these players were male (91.3%;115/126), with football players accounting for 102 of the 126 (81%) transient brachial plexus injuries. There have been many adjustments and rule changes in collegiate sports in recent years to lower the incidence of cervical spine injuries, although nonspecific to UENVIs. In football, the NCAA has introduced regulations to reduce the incidence of head-down contact and spearing, yet the incidence of cervical injury remains of significant concern.^15^ This highlights the need for team physicians and other health professionals to educate players and coaches on proper contact technique.

TOS and neurologic neck injuries were more likely to require physician evaluation, diagnostic testing, and surgical intervention compared to transient brachial plexus injuries. These players require prompt and comprehensive evaluation to facilitate timely intervention and optimize outcomes. In our study, most TOS cases were in female athletes (61.6%; 45/73), and most neurologic neck injuries were in male athletes (85.7%; 12/14). This highlights the unique challenges players faced based on sex and sport and the need for an individualized approach.

While most athletes in our study returned to their previous level of activity, a substantial proportion returned to play with restrictions or did not return to play at all. This underscores the potential long-term implications of these injuries on athletic performance and career longevity. The variability in time to return to play is likely due to the variable nature of these injuries and the variance in resolution of symptoms.

The present study has limitations. The retrospective nature of our study and the use of a single database introduce selection bias and reduce generalizability. However, to our knowledge, this is the first study to look at a wide variety of injuries across all sports in a large conference of NCAA athletes. Our study lacks specific information regarding treatment and return-to-sport algorithms. This heterogeneity leads to variability in how athletes with similar injuries are treated and when they return to sport. This limits the applicability of our findings to a specific player undergoing a specific treatment regimen but provides valuable general information about when an athlete may expect to return to play. Our analysis was also limited by the lack of total athlete population data, baseline demographic distribution, and detailed medical history among all Pac-12 athletes. We could not identify the true incidence of UENVIs across sports or determine whether certain racial or sex-based disparities exist at a population level. Future studies should aim to include comprehensive population data to allow for more accurate incidence and demographic analysis.

Lastly, further longitudinal studies and following collegiate athletes into their professional careers may provide more valuable insight into the effects of UENVIs on long-term performance and career longevity.

## Conclusion

Our study highlights the significant burden of UENVIs in collegiate athletics. These injuries affect athletes across a multitude of sports, emphasizing the need for comprehensive injury prevention strategies and early evaluation and treatment to optimize outcomes in affected athletes. Most athletes with UENVIs return to sport at the same or similar level of play. To our knowledge, this is the first study to report on race and sex in UENVIs in a large NCAA sports database. When football players are excluded, female athletes have a higher incidence of UENVIs. Further longitudinal studies following athletes into their professional careers are needed to fully explore the effect of UENVIs on long-term performance and career longevity.
